# Prostatectomy and Bladder Stone

**Published:** 1908-02-01

**Authors:** Reginald Harrison

**Affiliations:** Past Vice-President and Hunterian Professor, Royal College of Surgeons, England.


### Hospital Clinics.

### PROSTATECTOMY AND BLADDER STONE.

By REGINALD HARRISON, F.R.C.S., Past Vice-President and Hunterian Professor, Royal College of Surgeons, England.

Recent progress in the treatment of the enlarged prostrate by supra-pubic prostatectomy, and the experience which is rapidly extending and accumulating will be found on analysis to throw considerable light on the prevention and treatment of stone in the bladder, more especially in reference to its recurrent form. This association of stone in elderly adults is a common one. More than half the males I have operated upon, over 60 years of age (not less than 500), for stone in the bladder had also some degree of prostatic hypertrophy. Enlargement of the prostate leads up to calculus formation in at least three ways:

1. By the trapping of small calculi and gravel of renal origin on their way outwards, and detaining them in the bladder, where they form nuclei for further increment by Rainey's process of molecular coalescence.

2. By providing a more or less suitable and permanent reservoir above the obstructing gland for undischargeable urine, which is liable to undergo ammoniacal and other decomposition, and thus to supply one or more elements for the production of a calculus, probably a phosphatic one.

3. By leading to the formation of independent and noncontractile sacs and pouches within the bladder which provide additional receptacles for decomposing urine.

Trapping kidney stones or crystalline concretions is the common origin of primary bladder stones. In the latter position they may attain considerable dimensions, and often present on section all the commoner varieties of stone that the urinary organs are capable of forming. Such trapping not infrequently happens in males accustomed to void calculi or gravel naturally until about 60 years of age is reached, when the prostate commences to enlarge and obstruct. A wrong construction is sometimes put on this cessation until bladder irritability is noticed.

In females calculi are for the most part of vesical, not renal origin, not infrequently connected with foreign bodies.

In an article I published, on the formation of stone by molecular coalescence,* about the time which immediately preceded the more general adoption of prostatectomy, I referred to an analysis of 101 consecutive cases of stone in the bladder in different males which I had recently treated by crushing on Bigelow's lines. Though out of this number only six deaths occurred, 23 patients had a recurrence of stone which necessitated at varying intervals one or more repeated operations. The average age of these 101 persons was 63 years. Of them, 17 had considerable enlargement of the prostate, whilst, in addition to these, 12 had pouched or saculated bladders; and the same number were more or less dependent on their catheters at the time of the first operation. Hence it may be concluded that out of 101 persons who suffered from bladder stone 41 had in addition an enlargement of the prostate. On the other hand, in younger and otherwise healthy males, without any form of urethral or prostatic obstruction, the liability to recurrence of stone in the bladder is rare, and explainable, when it does happen, by fresh descents from the kidney of a diathetic stone too large to escape from the bladder without assistance with the lithotrite and evacuator.

Nor, relative to the frequency of recurring stone, is my experience an exceptional one. In this way, and without any reflection upon surgical skill, lithotrity tended to become discredited, and supra pubic lithotomy bade fair again to supplant Bigelow's historical operation. Prostatectomy has practically changed all this. Recurrence of bladder stone complicated with an enlarged prostate or its effects, may now be regarded as a good and sufficient reason in itself for the removal of the gland at the same time as the stone.

Within the last six years, that is to say, since my paper was published, I am cognisant of over 30 cases of bladder stone in which the prostate has been removed for symptoms such as an enlarged gland is capable of producing, combined with a recurrent stone following one or more of my previous operations by crushing.

I am not aware of any death happening when prostatectomy was undertaken for recurrent stone associated with prostatic symptoms. This circumstance was probably due to my good fortune in only having to deal with instances of adenomatous prostate which shelled out with comparative ease. When this is not the case, and the gland has to be broken up and removed piecemeal with the finger or forceps, the difficulty is considerably increased, and the possibility of malignancy has to be taken into consideration relative to prospective events, though the immediate and temporary effects of the operation may fully justify its performance. Carcinoma of the prostate is not prohibitory of prostatectomy. Having regard to the function of these parts, and the need and degree of relief that is required in some instances, it may prove the best alternative whether or not a stone is present and may be advised on this ground alone.

Some of my cases of adenomatous prostates with stone recurrences had remarkable histories. In one of the persons referred to lithotrity was repeated by me 13 times in the course of a few years. The patient got so used to this process that three or four days in a surgical home sufficed to clear him of a considerable mass of phosphatic stone without occasioning him any further detention and with complete temporary relief of varying duration.

Similarly two other cases each previously underwent lithotrity for recurrence of stone ten times, another six, and others fewer times. All eventually were submitted to prostatectomy, and though some

* *The Lancet*, Feb. 9, 1901.

years have elapsed since the latter operation I am only aware of one having had any recurrence of stone, and this occurred four years after the prostate was removed. In the meantime he, as well as the others, enjoyed good health without occasion to use a catheter. A record such as this is in itself sufficient to indicate without discussion that an enlarged prostate may prove a direct cause of stone in the bladder.

How far prostatectomy is applicable to primary cases of stone complicated with an enlarged prostate causing symptoms of its own is a matter which can only be considered in individual instances. Many examples have occurred where this has been successfully done, though in some cases the stone or stones were not discovered until the bladder was opened for the purpose of enucleating and removing the enlarged prostate. Hence the feasibility of the double operation has been sufficiently illustrated. On the other hand, it must be remembered that in a considerable proportion of cases in elderly males the enlarged prostate only asserts itself symptomatically so long as the bladder contains a stone. After this is removed all may remain quiet and natural. Where there seems a reasonable probability of this, lithotrity and evacuation is the simplest expedient with the least risk. If, in spite of this anticipation, recurrence of stone follows, then the prostate may also be removed.

Should, however, a concurrence of painful symptoms exist in which stone, bladder, prostate and catheterism, are all more or less concerned, a strong case is made out for a primary prostatectomy. In these circumstances, to get rid of such a combination is a consummation worth taking some degree of risk to obtain, and is quite within reach. Even persons of a very advanced age are often more likely, to use a common expression, to bear the operation well than the distressing symptoms which otherwise they would be called upon to endure.

Then there is this consideration, apart from the inconvenience connected with the life-long use of a catheter. The restoration of the natural function of micturition, which may usually be relied upon, is the greatest safeguard against the recurrence of stone and the mechanical disorganisation of the bladder by sacs and pouches which is often largely responsible for its production.

